# Transfer Learning in Multiple Hypothesis Testing

**DOI:** 10.3390/e26010049

**Published:** 2024-01-04

**Authors:** Stefano Cabras, María Eugenia Castellanos Nueda

**Affiliations:** 1 Department of Statistics, University Carlos III of Madrid, 28903 Madrid, Spain; 2 Department of Informatics and Statistics, Rey Juan Carlos University, 28933 Mostoles, Spain; maria.castellanos@urjc.es

**Keywords:** bayes factors, deep learning, improper priors, objective bayesian inference, random sequences, RNA-seq experiments, 62F15

## Abstract

In this investigation, a synthesis of Convolutional Neural Networks (CNNs) and Bayesian inference is presented, leading to a novel approach to the problem of Multiple Hypothesis Testing (MHT). Diverging from traditional paradigms, this study introduces a sequence-based uncalibrated Bayes factor approach to test many hypotheses using the same family of sampling parametric models. A two-step methodology is employed: initially, a learning phase is conducted utilizing simulated datasets encompassing a wide spectrum of null and alternative hypotheses, followed by a transfer phase applying this fitted model to real-world experimental sequences. The outcome is a CNN model capable of navigating the complex domain of MHT with improved precision over traditional methods, also demonstrating robustness under varying conditions, including the number of true nulls and dependencies between tests. Although indications of empirical evaluations are presented and show that the methodology will prove useful, more work is required to provide a full evaluation from a theoretical perspective. The potential of this innovative approach is further illustrated within the critical domain of genomics. Although formal proof of the consistency of the model remains elusive due to the inherent complexity of the algorithms, this paper also provides some theoretical insights and advocates for continued exploration of this methodology.

## 1. Introduction

Multiple hypothesis testing (MHT) is a collection of statistical methods that are used to perform more than one statistical test simultaneously. Specifically, if there are *m* statistical tests to be performed, these methods aim to categorize these *m* tests into two groups: $m_{0}$ tests where the null hypothesis is true, indicated as $H_{0}$ , and $m_{1}=m-m_{0}$ tests where the alternative hypothesis is true, indicated as $H_{1}$ . This categorization is conducted in a way that controls the maximum allowable value of some error. MHT methods are particularly useful in applied statistics because they can handle large datasets, such as Big Data, where the real problem at hand often involves testing multiple scientific hypotheses simultaneously. One of the pioneering applications for MHT methods is in Genome-Wide Association Studies (GWASs) using RNA-seq technology. In this context, *m* represents the number of gene abundance tests for an Expression Sequence Tag (EST) in a given biological sample. These abundance counts are then compared across two different biological populations to test *m* hypotheses about comparisons and identify genes associated with a specific characteristic or phenotype related to the two biological populations.

Because it is not feasible to model all tests together using a single comprehensive sampling model, each test (each gene) is modeled independently. These individual models are combined in an MHT procedure. The MHT procedure takes the marginal evidence from each test as input and outputs the joint evidence in all tests for a given threshold *t* in all tests which leads to a separation between tests supposed to be under $H_{0}$ from those under $H_{1}$ . The primary goal of using an MHT procedure is to control certain types of errors. On the one side those related to the number of errors in declaring tests as coming from $H_{1}$ when they are actually from $H_{0}$ : these are the False Positive Rate ( FPR(t) ) the proportion of false rejections among all rejections and the False Discovery Rate (FDR), which is the expectation over the random sample of FPR(t) , it is the proportion of those rejections that should not have been rejected; while on the other side, we wish to control the proportion of missed rejections (declaring tests as coming from the $H_{0}$ when they are from $H_{1}$ ) overall rejection, the False Nonrejection Proportion ( FNP(t) ) and its expectation False Non-rejection Rate (FNR). An MHT procedure aims to generate a Receiver Operating Characteristic (ROC) curve: 1−FPR(t) versus FPR(t) so that the Area Under the Curve (AUC) approaches 1. This happens when FPR(t)→0 for all thresholds *t* and considering the expectation referred to above, this is equivalent to having a FDR→0 . In this sense, we consider equivalent controlling FPR and FDR, although they are not exactly the same. This occurs, for example, for the well-known Benjamini–Hochberg (BH) procedure [1] for m→∞ under assumptions untestable on a given sample [2]. These assumptions generally relate to the dependencies between the tests and the marginal sampling distributions of the test statistics [3], which are represented by the *p*-values $pv_{1},\ldots ,pv_{m}$ .

The most common MHT methods usually rely on *p*-values generated by statistical tests. Because *p*-values can be derived from a wide range of statistical models, from simple to complex models such as Bayesian models with intractable likelihoods [4], they are often the go-to choice in MHT. In ideal conditions where the null hypothesis is simple or the test statistic is ancillary to the nuisance parameters, the *p*-values are uniformly distributed between 0 and 1. Such *p*-values are referred to as *calibrated*, making constructing reliable MHT procedures straightforward.

However, these ideal conditions are rare. In many practical cases, the *p*-values deviate from the uniform distribution, making them unreliable for controlling the FDR [3].

This problem has led researchers to create adaptive MHT methods that estimate the true distribution of *p*-values [5,6,7]. There is also growing interest in using additional samples to recalibrate existing MHT methods [7,8]. We propose a contemporary approach that utilizes deep learning techniques for this recalibration. The way we use this technique is also known as *transfer learning* in the machine learning language. Transfer learning is meant as a statistical technique in machine learning where a model developed for a particular task (i.e., analyzing an MHT problem where the truth is known) is reused as the starting point for a model on a second task (i.e., analyzing an MHT problem where the true is unknown). It is an optimization that allows for rapid progress or improved performance when modeling the second task. In the context of neural networks, this involves transferring the weights and learned features from a pre-trained neural network to a new neural network being trained for a different, but related, task.

Here, to work around the difficulties of using *p*-values that are often uncalibrated, we call for a Bayesian approach using Bayes factors (BF) for testing. BFs are the ratio between the marginal probability of the sample under the alternative hypothesis and the null. Individual test evidence from a BF strongly depends on the prior evidence for the unknown parameters in the composite null hypotheses. These are parameters that are not common to all hypotheses, and the prior distribution affects the marginal of the data and hence the BFs. Then the BFs could be arbitrarily driven by the prior rather than by the data. In contrast, for MHT, it has been shown in [9] that it is possible to use the so-called uncalibrated BF, $B_{i}$ , from $cB_{1},\ldots ,cB_{m}$ , where cBi is the ith full BF of the alternative hypothesis against the null in test i, and c > 0, is the ratio between the two prior normalization constants for the null and alternative hypotheses. $B_{i}$ is not a BF as it misses the prior normalizing constants *c* which is why it is referred to as uncalibrated, i.e., its interpretation is not that of the relative evidence between two hypotheses. Suppose that many tests involve models with one or more nuisance parameters (composite null hypotheses). In this case, an expert would need to elicit a prior distribution of unknowns in each test, which is unfeasible given the large value of *m*. Therefore, substituting the presence of an expert by employing formal rules in the prior definition leads to two usually vague or improper priors that must be employed for the alternative and null models. Therefore, the BF for the single hypothesis test is not determined due to the ratio between the prior *pseudo*-constants $c=c_{1}/c_{0}$ , where $c_{0}$ and $c_{1}$ are the unbounded prior normalization constants for the parameters of the null hypothesis, $H_{0}$ and the alternative $H_{1}$ , respectively (see [9,10]). Furthermore, in [9,10], it is shown that the use of proper well-calibrated priors, leading to fully defined and interpretable BFs $cB_{i}$ , is not necessary in MHT. Uncalibrated BFs, $B_{i}$ in MHT avoids employing nonscalable computational techniques to obtain a properly defined BF $cB_{i}$ for each test.

Although a comprehensive overview of the literature on Multiple Hypotheses Testing (MHT) is beyond the scope of this article, readers are encouraged to consult seminal review articles such as [11,12,13] for insights specifically relevant to Genome-wide Association Studies (GWAS).

The crucial insight to highlight is that all MHT methodologies essentially rely on an ordered sequence of test statistics, and the order is sample-dependent, and thus per se random. This is typically presented as $pv_{1}\geq pv_{2}\geq \cdots \geq pv_{i}\cdots \geq pv_{m}$ in conventional methods or as $cB_{1}\leq cB_{2}\leq \cdots \leq cB_{i}\cdots \leq cB_{m}$ in the BF approach [9,10], in which it is clear that c>0 does not alter the order. These ordered sequences serve as the output of various statistical tests and the input of MHT and the methodology proposed in this article. The sequences are valuable for segregating the tests that fall under the null hypothesis from those that fall under the alternative hypothesis.

The core concept is that the same sampling models used for obtaining *p*-values or BFs can also produce a training set of either *p*-values or BFs. The labels for the null and alternative hypotheses under which *p*-values or BFs are generated are already known in this training set. This training set can then be used to fit a classification model during a learning phase. In this light, the problem of MHT becomes one of classifying subsequences of *p*-values or BFs originating from either the null or alternative hypotheses.

Today, the problem of classifying subsequences is very well handled using complicated functions known as Neural Networks (NN), for instance, the *one-channel* convolutional NN (CNN). CNN is a specific class of NN suitable for analyzing structured (e.g., dependent) samples as images [14] with the general purpose of classifying them to obtain, for example, a medical diagnosis [15]. Furthermore, for our purposes, it is worth stressing that there is evidence that CNNs are very useful for analyzing Time Series [16] better than other architectures such as NN recurrent and long-short-term memory [17]. The architecture of a CNN is based on a connecting set of neurons (mathematical operations on some input) that are supposed to be trained to recognize relevant features in the observed sequence to achieve the minimum classification error, and thus the minimum FPR and FNP.

In the following sections, we limit our discussion to those aspects of CNNs directly relevant to their application in MHT. We refer the reader to existing reviews or books on the subject for a more comprehensive understanding, such as [14]. The rest of this paper provides the actual code (based on Keras and Tensorflow) used to construct and train CNN. Detailed technical information is available there, and we conclude by emphasizing that all computations were performed on a standard laptop, negating the need for specialized hardware. The accompanying R code is accessible at: 14 December 2023 https://colab.research.google.com/drive/1TdM1FSVKm1GI55riUXoLbzcEcM3FeoNg?usp=sharing.

The remainder of this paper is structured as follows. Initially, Section 2 outlines the MHT approach that we propose, with particular emphasis on estimating the probability, denoted $\hat{p}$ , that a hypothesis is part of the alternative set. Subsequently, Section 2.1 demonstrates this framework through a simulation study based on a parametric example. Subsequently, Section 4 delves into more intricate models by revisiting two RNA-seq experiments, where we contrast the evidence derived from BF and conventional *p*-values. Concluding thoughts with an exposition of the limitations of this approach and additional comments are reserved for Section 5.

## 2. Convolutional Neural Network for Multiple Testing Arising from Uncalibrated Bayes Factors

The MHT methodology elaborated in this study is based on a sequence of ordered BFs, $cB_{i}$ , i=1,…,m , ranging from the weakest to the strongest evidence of some common alternative hypothesis (to *m*) against a common null hypothesis. This empirically determined sequence serves as the classification subject for CNN.

To be precise, consider $cB_{0}=cB_{1}\leq cB_{2}\leq \ldots \leq cB_{m}$ as an ordered set of m+1 BFs, where $cB_{0}$ is only introduced for notational convenience. We define the relative weights of the evidence among the *m* alternative hypotheses as

$$W=\left\{W_{i}=\log\left(cB_{i}/cB_{i-1}\right),\;\mathrm{for}\;i=2,\ldots ,m\right\},$$

with $W_{1}=1$ . This set W represents the differential evidence that favors each alternative hypothesis relative to its predecessor.

The ordered sequence of interest is $w=(w_{1},\ldots ,w_{m})$ , where each $w_{i}$ corresponds to a BF $cB_{i}$ . Tests appearing earlier in this sequence are supposed to originate from the null hypothesis, while those appearing later are more likely from the alternative. In particular, although the individual $cB_{i}$ may not be interpretable due to the unknown scaling factor *c*, the relative evidence $W_{i}$ remains meaningful [9].

Let the original set of hypotheses, denoted as H , be partitioned into two supposed nonempty sets $H_{1}$ and $H_{0}$ , representing true alternative and true null hypotheses, respectively. To estimate $H_{1}$ with an ordered sequence of *W*, it would suffice to estimate its size $m_{1}$ , subject to $1\leq m_{1}\leq m$ . As discussed in Section 3, this can often, but not necessarily, be accomplished by estimating the position $\hat{i}\in \left(1,\ldots m\right)$ of a change point in the sequence w .

**Figure 1:**
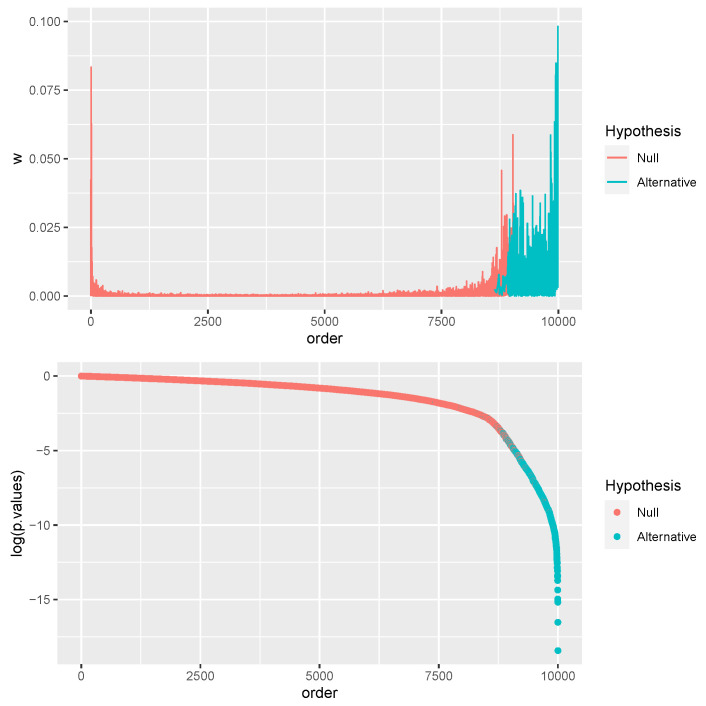
(**top**) A graphical representation of the sequence $W_{1},\ldots ,W_{m}$ (vertical) along ordered tests (horizontal) used for training the CNN for subsequent predictions concerning $H_{1}$ and $H_{0}$ . (**bottom**) Corresponding *p*-values (vertical) along ordered tests (horizontal) from *t*-Student tests with Welch’s correction.

To elucidate, Figure 1 shows a simulated example with tests m=10,000 and $m_{1}=1000$ , detailing how this methodology works in practice.

Figure 1 also reports the already mentioned training set for CNN that will be applied later in real-world scenarios where $H_{1}$ and $H_{0}$ are unknown.

The efficacy of using this particular CNN formulates the crux of our argument, affirming that the method’s robustness is not heavily dependent on various unknowns such as the number of tests under the null, $m_{0}$ , data signal strength (i.e., sharp or no sharp evidence from tests), test dependencies, and sampling distributions, among others.

### 2.1. Sequence of the Relative Weights of Evidence

This section introduces a generalized definition of W customized later for specific parametric model environments. Consider a vector of experimental outcomes, $x=(x_{1},\ldots ,x_{m})$ , each featuring *m* distinct attributes such as gene abundance levels measured by RNA-seq counts. The vector $x_{i}$ includes $n_{i}$ replications for the *i*th feature, with i∈(1,…m) .

The MHT issue can be framed as a Bayesian multiple model selection problem, where each test *i* compares the evidence supporting the alternative hypothesis $H_{i1}$ against the null hypothesis $H_{i0}$ , which have the same prior probabilities (i.e., $P\left(H_{i1}\right)=P\left(H_{i0}\right)=1/2$ ) as follows:

(1) $$\begin{array}{cc} H_{i0} & :f_{i0}\left(x_{i}|\theta_{i0}\right),\;\pi_{i0}\left(\theta_{i0}\right),\;\theta_{i0}\in \Theta_{i0},\\ H_{i1} & :f_{i1}\left(x_{i}|\theta_{i1}\right),\;\pi_{i1}\left(\theta_{i1}\right),\;\theta_{i1}\in \Theta_{i1}, \end{array}\;i=1,\ldots ,m.$$

Here, $\pi_{i0}\left(\theta_{i0}\right)$ and $\pi_{i1}\left(\theta_{i1}\right)$ are typically default and often improper prior distributions on the unknown model parameters. The sets $\left\{\Theta_{i0},\Theta_{i1}\right\}$ are a of partition $\Theta_{i}\subset {ℜ}^{K}$ , where K≥1 .

Prior distributions are assumed to be the same across all tests (as the sampling model in each test) and are derived from a standard formal rule applied to each $f_{ik}\left(\cdot |\cdot \right)$ for k=0,1 . Such rules include but are not limited to, Jeffreys, Intrinsic, Reference, Matching, Nonlocal priors, or Conventional priors [18,19,20,21,22,23].

Consequently, for all *i*:

(2) $$\begin{array}{cc} \pi_{i0}\left(\theta_{i0}\right) & =\pi_{0}\left(\theta_{0}\right)\propto c_{0}g_{0}\left(\theta_{0}\right),\\ \pi_{i1}\left(\theta_{i1}\right) & =\pi_{1}\left(\theta_{1}\right)\propto c_{1}g_{1}\left(\theta_{1}\right), \end{array}$$

where $g_{0}$ and $g_{1}$ are two positive functions (not necessarily measurable) and $c_{0}$ and $c_{1}$ act as normalizing pseudoconstants.

We assume the existence of prior predictive distributions for both the null and alternative hypotheses.

(3) $$m_{ik}\left(x_{i}\right)=\int_{\theta_{k}\in \Theta_{k}}f_{k}\left(x_{i}|\theta_{k}\right)\pi_{k}\left(\theta_{k}\right)d\theta_{k},\;\mathrm{for}\;k=0,1,\;i=1,\ldots ,m.$$

The BF of $H_{i1}$ against $H_{i0}$ can then be formulated as:

(4) $$cB_{i}=\frac{m_{i1}\left(x_{i}\right)}{m_{i0}\left(x_{i}\right)}=\frac{c_{1}}{c_{0}}\cdot \frac{\int_{\theta_{1}\in \Theta_{1}}g_{1}\left(\theta_{1}\right)f_{1}\left(x_{i}|\theta_{1}\right)d\theta_{1}}{\int_{\theta_{0}\in \Theta_{0}}g_{0}\left(\theta_{0}\right)f_{0}\left(x_{i}|\theta_{0}\right)d\theta_{0}},$$

This calibrated BF, in the sense that it reports the posterior relative evidence of $H_{i1}$ against $H_{i0}$ according to the Jeffreys interpretation, is practically unscaled due to the arbitrarily low ratio $c=c_{1}/c_{0}>0$ . We then define the uncalibrated or unscaled BF as follows:

(5) $$B_{i}=\frac{\int_{\theta_{1}\in \Theta_{1}}g_{1}\left(\theta_{1}\right)f_{1}\left(x_{i}|\theta_{1}\right)d\theta_{1}}{\int_{\theta_{0}\in \Theta_{0}}g_{0}\left(\theta_{0}\right)f_{0}\left(x_{i}|\theta_{0}\right)d\theta_{0}}.$$

Although $B_{i}$ lacks standalone interpretability, it serves as a comparative measure [9,10]. For example, if $\exp\left(W_{i}\right)=\frac{B_{i}}{B_{i^{\prime }}}>1$ for all $i,i^{\prime }$ , then the evidence supporting $H_{i1}$ over $H_{i0}$ is stronger than that for $H_{i^{\prime }1}$ over $H_{i^{\prime }0}$ , regardless of *c*.

In summary, even if the priors are specified as vague or improper, their normalizing constants are effectively simplified in the sequence of *W*s. This does not imply that priors are irrelevant in MHT, but their impact, specifically that of the constants $c_{0}$ and $c_{1}$ , is mitigated in the collective evidence derived from the tests.

### 2.2. Convolutional Neural Network on the Sequence of Relative Weights of Evidence

A one-dimensional CNN is fitted to the observed sequence w . The loss function used is the binary cross-entropy, which is the logarithmic representation of the Bernoulli density: $\sum_{i=1}^{m}\log({\hat{p}}_{i}^{H_{i}}{\left(1-{\hat{p}}_{i}\right)}^{1-H_{i}}$ , where $H_{i}=1$ ( $H_{i}=0$ ) if the test *i* has been observed under the alternative (null) in the training set. This function minimizes the classification error, and thus the FPR and FNP. For each test *i*, the fitted CNN produces a point estimate of the probability, ${\hat{p}}_{i}$ , that it belongs to $H_{1}$ . The decision about the set of observed tests from the alternative is formulated as

$${\hat{H}}_{1}=\left\{i:{\hat{p}}_{i}>1-q\right\},$$

where *q* is the FDR level (or the averaged FPR) that we want to control when testing the hypotheses. It can be argued that ${\hat{p}}_{i}$ is the maximum *a posteriori* probability that the test *i* is observed under the alternative given the set of *m* tests and the priors of the underlying Gaussian process prior to the NN weights [24].

Using CNN, we establish a complicated function $\mathrm{CNN}:w\mapsto \hat{p}=\left({\hat{p}}_{1},\ldots {\hat{p}}_{m}\right)$ that accounts for the dependencies among the *W*’s. This is how the *m* tests are jointly considered to control the FDR at level *q*, as will be discussed in the next Section 3.

The assumption of exchangeability between the training (simulated) sample and the observed sample of *W*’s is understood within the context of the sampling model induced by the fitted CNN. The existence of untestable assumptions needed when using the usual MHT has to be compared with the possibility of assessing the goodness-of-fit of a trained CNN. This can be conducted using routine analyses typical of the machine learning literature by assessing performance on test sets, which can also be simulated. This is exactly what is conducted in Section 4.2. In general, the fact that in Section 4.2 the sampling model for individual tests differs from the one used in the training set lends reliability to the estimates $\hat{p}$ even if CNN was fitted only to a simulated data set of m=10,000 , $w^{*}$ , as shown at the top of Figure 1. For this purpose, it is crucial to note that we trained only one CNN in $w^{*}$ in Figure 1 for all subsequent analyses in this paper. In the training sample, we have m=10,000 values of $w^{*}$ simulated from independent tests on the mean of two independent normal populations with equal but unknown unit variances, as detailed in Section 4.2. In $w^{*}$ , we have $m_{1}=1000$ tests simulated from $H_{1}$ with a mean 3 in one population and the rest from $H_{0}$ with both populations with zero mean.

**Figure 2:**
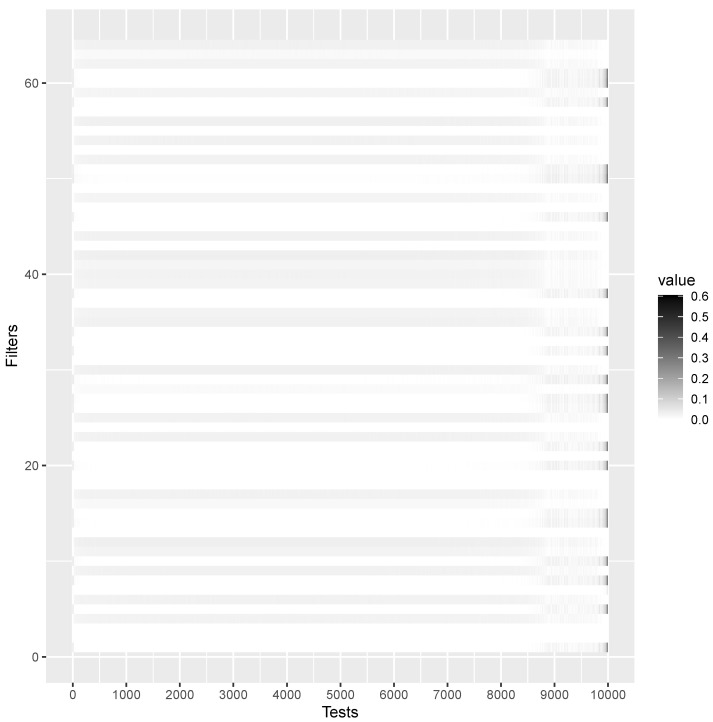
Feature map induced by the first convolution layer, which has 64 filters (rows) on all tests (columns) for the CNN fitted on the sequence of *W*’s in Figure 1. The higher the activation, the darker the cell. Estimated point $\hat{i}$ is around 9001 as $m_{0}=9000$ .

Understanding CNN from the MHT perspective is important for confidence in the proposed method. A CNN is an NN where (deterministic) nodes are functions of inputs and are connected according to a specific structure. Nodes are typically operations with weights that are set to minimize the global error in classifying the results of tests in $H_{1}$ (or $H_{0}$ ) when they come from $H_{0}$ (or $H_{1}$ ). In a CNN, we have two types of nodes:

1. *Feature detection nodes.* They have as input the subsequences of w ,
$${\tilde{w}}_{i,k}=\left(w_{i},w_{i+1},\ldots ,w_{i+k-1}\right),$$
  where k≥2 is known as the *kernel size*. These subsequences of a minimum length of w bear local information about the random sequence of *W*’s. Such nodes return subsequences of $\tilde{w}$ ’s all of the same size *k*, in which relevant features are detected through the so-called *filters*. Filter functions are defined on sets of weights and are devoted to detecting *locally* features on each ${\tilde{w}}_{i,k}$ . The systematic application of the same filter across the $\tilde{w}$ sequences is useful for our problem. Each filter, that is, each set of weights, detects a specific behavior in the series w , especially near the separation point $W_{\hat{i}}$ , mentioned above. The problem is that we do not know the relevant behavior to be detected and where it should be expected in the *W*’ s sequence. Therefore, all filters are applied to all sequences $\tilde{w}$ , along the entire observed sequence of *W*’s. This filtering process allows CNN to discover what and where the behavior of *W* is expected to estimate $\hat{p}$ correctly. We know, for example, that it should be important to analyze the behavior around $W_{\hat{i}}$ where $\hat{i}$ can be anywhere between 1 and *m*. This capability is commonly referred to as the *translation invariance* of a CNN. Fortunately, and contrary to the usual interpretation of NN as black boxes, it is possible to show the features detected by each filter, as shown in Figure 2, which reports the CNN weight values for the first convolution layer, which has 64 filters and thus weights 64×m . From Figure 2, it is possible to appreciate a change of activation around position 9001, which is the actual $\hat{i}$ point mentioned above.
2. *Pooling nodes*. These nodes connect all filters through the *pooling function*. The filter is just a dot product of the input, ${\tilde{w}}_{i,k}$ , using a set of weights. The output of such a product is the input of the pooling function that leads to a result of a dimension less than *k*, for example, considering the maximum output resulting from a filtered ${\tilde{w}}_{k,i}$ . The set of pooling nodes is also called the *feature map* because it gives a map of the relevant filters along the sequence of *W*’s for classifying tests.

The architecture of the CNN captures three vital characteristics when modeling w :

- *Localized Feature Detection:* Kernels of small sizes are employed to focus on local features in the series of w ’s. This contributes to sparse modeling of the sequence and enables the capture of intricate dependencies between tests.
- *Parameter Efficiency:* To achieve parsimonious modeling, the same set of weights (i.e., model parameters) are reused throughout the sequence w . This design leverages the power of shared evidence for NN parameters, offering a more accurate weight estimation based on multiple samples.
- *Robust Feature Recognition:* The CNN is equipped to identify critical features in the data sequence, invariant to factors such as location, scale, position of the separation point $\hat{i}$ , and test dependencies. This robustness potentially uncovers features to be described that are instrumental in estimating the sets $H_{0}$ and $H_{1}$ . For example, the translation invariance property mentioned above is not shared by common change point detection techniques [25], such as the cumulative sum control chart (CUSUM) [26].

Further details about the specific CNN architecture used in this study are provided in Appendix B. Although we do not insist that this architecture is universally optimal for MHT, it has been proven effective for the illustrative purposes of this paper.

To reiterate, we trained a single CNN model using the simulated set of *m* tests displayed in Figure 1. Subsequent results validate CNN’s capability for MHT, demonstrating its proficiency in classifying tests. For other practical scenarios, CNN could be trained using the results of a calibration experiment—assuming that such data are available and the ground truth is known—instead of relying on simulated data as in Figure 1.

## 3. Sketch of the Theory

The objective of this section is not to provide rigorous proof of the method’s consistency but to offer theoretical insights supporting its asymptotic behavior, as observed in the simulation studies. Specifically, our objective is to theoretically substantiate that the proposed method demonstrates asymptotic consistency with respect to both the sample size *n* and the number of tests *m*, as evidenced by negligible FDR and FNR for sufficiently large values of *n* and *m*.

First, at a specific separation point $\hat{i}$ in the sequence of ordered Bayes factors $B_{i}$ , the corresponding $W_{\hat{i}}$ is defined as:

$$W_{\hat{i}}=\log\left(\frac{\mathrm{Pr}(Test\;at\;\hat{i}\in H_{1}|W)}{1-\mathrm{Pr}(Test\;at\;\hat{i}\in H_{1}|W)}\right)-\log\left(\frac{\mathrm{Pr}(Test\;at\;\hat{i}-1\in H_{1}|W)}{1-\mathrm{Pr}(Test\;at\;\hat{i}-1\in H_{1}|W)}\right),$$

where $\mathrm{Pr}(Test\;at\;\hat{i}\in H_{1}|W)$ indicates the marginal probability of observing the evidence of test $\hat{i}$ in the alternative set, which is the numerator of BF $cB_{\hat{i}}$ .

The proposition guarantees the asymptotic existence of this separation point $\hat{i}$ in the sequence of *W*’s.

**Proposition 1.**  *For n→∞ then $W_{\hat{i}}\to \infty$ .*

**Proof.**  The proof relies on the well-known consistency of BF (see, e.g., [27]) for every 0<c<∞ , that is, for n→∞ and $i\neq \hat{i}$ , we have $cB_{i}\to 0$ (for $i<\hat{i}$ ) or $cB_{i}\to \infty$ (for $i>\hat{i}$ ) and thus $W_{i}\to 0$ for $i\neq \hat{i}$ . At the separation point $\hat{i}$ we have $cB_{\hat{i}}\to \infty$ as $\hat{i}\in H_{1}$ and $cB_{\hat{i}-1}\to 0$ as the test $\hat{i}-1\in H_{0}$ , therefore, $W_{\hat{i}}=cB_{\hat{i}}/cB_{\hat{i}-1}\to \infty$ .    □

Second, the objective of the paper is to present evidence supporting the asymptotic consistency of the CNN estimator $\hat{p}$ as m→∞ . Previous work [28,29], has established the consistency of feedforward NNs in distance $L_{2}$ , which can, in principle, be applied to CNNs, although there is no specific literature on CNN consistency [30,31]. Define $h_{0}\left(W\right)$ as a CNN oracle such that $h_{0}\left(w_{i}\right)=1$ if you test $i\in H_{1}$ and 0 otherwise. The CNN adjusted to the tests of *m* is denoted by $\hat{h}\left(W\right)$ . According to the aforementioned literature [28,29,32], the distance $L_{2}$ between these CNNs vanishes asymptotically as m→∞ :

$$\int {\left(h_{0}\left(W\right)-\hat{h}\left(W\right)\right)}^{2}dW\to 0.$$

Furthermore, we argue that $h_{0}\left(w_{i}\right)\to j$ is as n→∞ for each $i\in H_{j}$ , where j∈{0,1} . This is a less restrictive condition than assuming the same stochastic process generating the observable variables, and thus the BFs. It is well known that the asymptotic consistency of the BFs can be achieved in the true model, but as *n* increases, the BF favors the model (although not the true one), minimizing the Kullback–Leibler (KL) divergence, making the model closest (to the true one) increasingly probable. CNN learns this characteristic of BF as illustrated in Figure 2, where CNN successfully identifies the characteristics around the discrimination point $\hat{i}$ .

In this framework, the CNN can accurately classify the tests $i<\hat{i}$ as belonging to $H_{0}$ and the rest to $H_{1}$ . The method offers bounded FDR and FNR as *n* and *m* grow. As mentioned above, the joint control of FDR and FNR suggests that the area under the ROC curve is AUC→1 .

Empirical validation supports AUC→1 , which confirms the robustness and effectiveness of the model.

We also applied the transfer learning approach to the *p*-values by repeating the same analysis on BFs, but we do not have evidence of high AUC as in the case of BFs.

## 4. Simulations and Real Examples

### 4.1. Training Dataset

The data set shown in Figure 1 is generated to address the classical statistical problem of testing the equivalence of means between two independent normal populations subject to heteroskedasticity. Specifically, we consider two independent populations, $X∼\mathrm{Normal}(\mu_{X},\sigma_{X}^{2})$ and $Y∼\mathrm{Normal}(\mu_{Y},\sigma_{Y}^{2})$ , each with sample sizes $n_{x}$ and $n_{y}$ , respectively. This example has been extensively detailed in [9,10], and additional information on computing the unscaled Bayes factor, $B_{i}$ , is provided in Appendix A.

**Figure 3:**
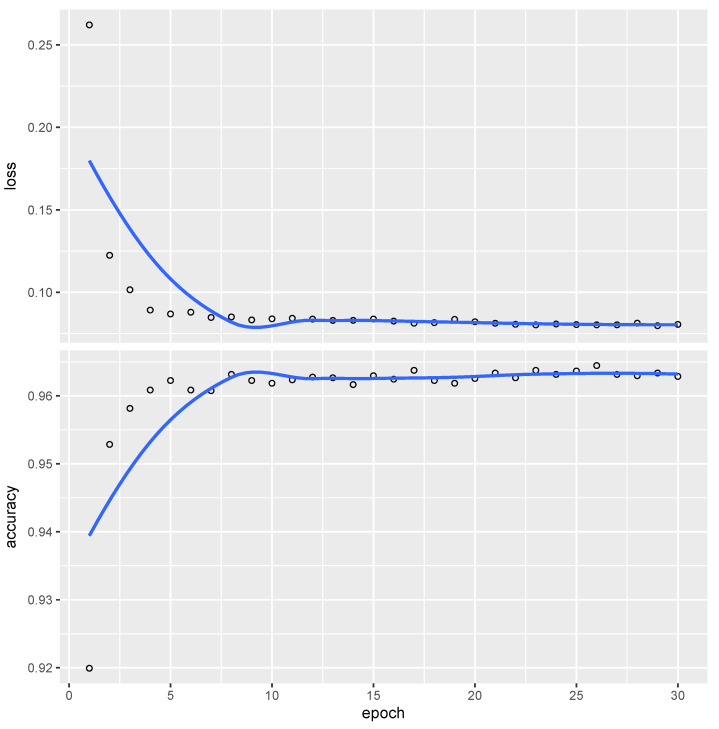
Evolution of error (top-vertical) and accuracy (bottom-vertical) for the CNN across the optimization epochs (horizontal), trained on the *W* sequence shown in Figure 1.

Figure 1 illustrates a sequence of *W*s generated with parameters $n_{x}=n_{y}=10$ , m=10,000 , $m_{0}/m=0.9$ , $m_{1}=1000$ , and distribution parameters set as specified. CNN underwent 30 optimization epochs, with the training result presented in Figure 3.

The trained CNN achieves an accuracy slightly exceeding 96%, indicating that fewer than 4% of the tests are misclassified relative to $H_{0}$ and $H_{1}$ in this large sample.

Furthermore, to assess the advantage of using *W*s over *p*-values for better hypothesis testing representation, as discussed in [9,10], we also trained the CNN on an ordered sequence of *p*-values, achieving comparable accuracy levels as shown in Figure 3.

In subsequent sections, we juxtapose our CNN-based approach with traditional methods commonly used in medical research. We compare the evidence derived from ordered *p*-values, obtained using Student’s *t*-test with Welch’s correction, to that obtained through our CNN model. These *p*-values are further adjusted using the BH FDR procedure, serving as our benchmark in the actual practice of MHT. Other procedures could have been considered [13,33,34], but keep in mind that, for instance, the BH procedure is also considered the limiting procedure of other approaches to MHT as the *q*-values when m→∞ [35]. Therefore, other approaches would not have added much to the exposed results.

We then evaluate these methods using the ROC curve to account for various experimental conditions in which different types of error may be of differing importance. The AUC serves as a summary metric to evaluate the precision in classifying the null and alternative hypothesis sets, $H_{0}$ and $H_{1}$ , thus controlling the corresponding FDR and FNR.

### 4.2. Simulation Study

Employing the pre-trained CNN with varying input features—specifically *W*s, *p*-values, and *p*-values adjusted according to the Benjamini–Hochberg (BH) scale—we systematically evaluate the average AUC through 1000 replications of the AUC. These replications are obtained under a composite set of scenarios designed to mirror various real-world conditions encountered in GWAS. The scenarios are delineated as follows.

- **Signal Variation for Alternatives:** The means for the alternative hypotheses, $\mu_{Y_{i}}$ , are set to values in the set {1,2,3,4} for $i\in H_{1}$ .
- **Sample Size and Asymptotic Behavior:** Both $n_{x}$ and $n_{y}$ are set to the value *n*, which ranges from 3 to 10.
- **Heteroscedasticity:** The standard deviations $\sigma_{X_{i}}$ and $\sigma_{Y_{i}}$ for i=1,…,m and $i\in H_{1}$ vary in the set {1,2,3} .
- **Proportion of True Alternatives:** The ratio $m_{0}/m$ is adjusted to one of the following: 0.9, 0.95, or 0.99.
- **Asymptotic Number of Tests:** The total number of tests, *m*, is set to 1000 or 10,000.
- **Test Dependence:** Two schemes are considered, one with all independent tests and another with block-dependent tests. In block-dependent tests, there are tests belonging to a set that are dependent on them and independent of the others, and there are also different sets. These tests induced a block-diagonal correlation matrix among the test statistics (see [36]). In the block-dependent case, variable blocks $X_{i}$ , $Y_{i}$ of sizes 2, 5, and 10 are formed, and their correlations are drawn randomly from a uniform distribution between −1 and 1, subject to the constraint of a positive semidefinite correlation matrix.

**Figure 4:**
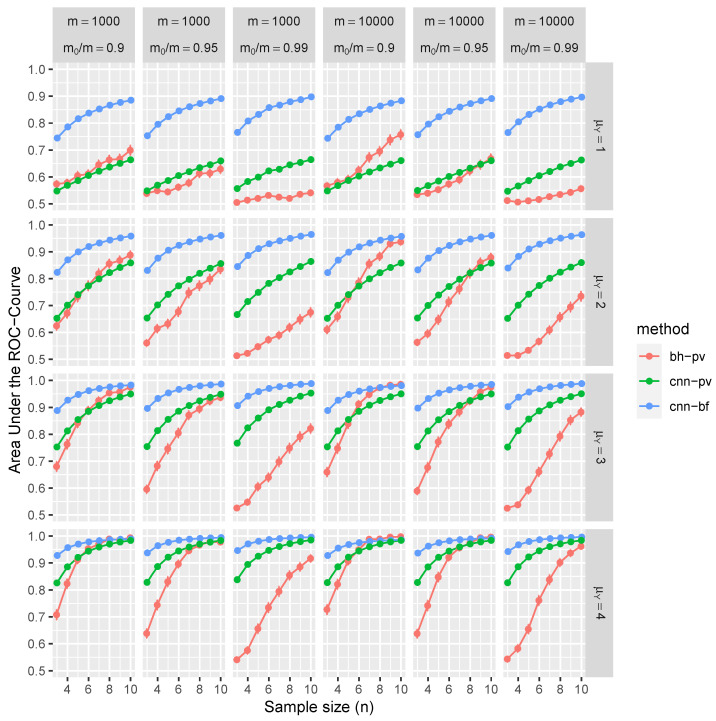
This figure illustrates the average AUC (depicted on the vertical axis) complemented by the corresponding 99.9% confidence intervals (barely visible as the average is over more than 1000 replications). These statistics are derived from independent tests under varying simulation conditions, such as sample sizes *n*, total number of tests *m*, and the proportion $m_{0}/m$ of null tests (denoted on the horizontal axis). Additionally, the mean of *Y* for tests under the alternative is represented on the right vertical axis. The average AUCs for the BH procedure (in red), the CNN based on *p*-values (in green), and the proposed CNN based on *W*s (in blue) are presented.

**Figure 5:**
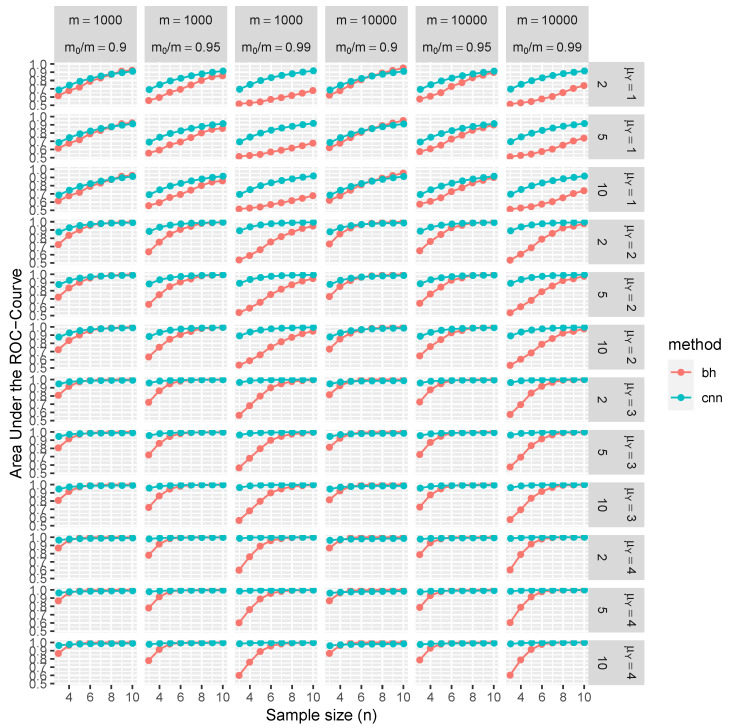
Similar to Figure 4, this figure portrays the average AUC and its 99.9% confidence intervals but for block-dependent tests. The averages are conditioned to: sample sizes *n*, total number of tests *m*, proportion $m_{0}/m$ of null tests (horizontal axis), and the mean of *Y* under the alternative and block sizes (denoted on the right vertical axis). The average AUCs for the BH procedure (in red) and the proposed CNN based on *W*s (in blue) are exhibited.

Figure 4 and Figure 5 present the statistical evaluation of the average AUC along with its 99.9% confidence intervals. These metrics are computed across 1000 replications and under various simulation scenarios explicitly enumerated in each figure’s axis labels and captions.

Consider the top-left panel of Figure 4 for illustrative purposes. It shows the average AUC and associated confidence intervals, conditioned on independent tests with parameters m=1000 , $m_{0}/m=0.9$ , $\mu_{Y_{i}}=1$ for $i\in H_{1}$ and *n* ranging from 3 to 10. Color-coded markers represent the BH procedure (in red), CNN applied to *W*s (in blue), and CNN also applied to *p*-values (in green). The results in this panel are marginal outcomes aggregated over 1000 replications and for all combinations of $\sigma_{X_{i}}$ and $\sigma_{Y_{i}}$ . Figure 5 extends this analysis to include block-dependent tests, which are also conditioned on the sizes of these blocks.

The results of our simulation study provide compelling evidence that the proposed CNN demonstrates robust control over FDR and FNR in a diverse range of conditions. Remarkably, these performance metrics are better than those achieved using BH procedures. Furthermore, CNN’s efficacy is noticeably higher when trained on test statistics *W* than when trained on classical *p*-values.

The distinctive advantages of the proposed methodology become more pronounced in scenarios with weaker signals, characterized by lower means ( $\mu_{Y}$ ), greater variances, or smaller sample sizes (*n*). These disparities can be attributed to the fact that the *p*-values are calibrated asymptotically in *n*, and the number of samples is often limited, especially given the high costs associated with replications of RNA sequences. Interestingly, the selected range of *m* has a negligible impact on CNN performance. On the contrary, traditional MHT procedures, such as the BH method, control FDR asymptotically in *m*, which is evident through slight improvements in the ROC curve at higher values of *m*. In summary, the increased dependencies among tests further accentuate the benefits of employing the CNN-based approach over traditional methods.

### 4.3. Example RNA-Seq 1: Squamous Cell Carcinomas versus Matched Normal Tissue

We examine RNA-seq count data from a paired design study focusing on oral squamous cell carcinomas and the corresponding normal tissue samples from six patients [37].

The primary objective of the analysis is to identify genes that exhibit differential expression between tumor and normal tissue samples. To account for patient-specific variations, we employ a mixed-effects Bayesian Poisson regression model with flat improper priors for the Poisson regression coefficients. Additionally, an exceedingly vague prior is utilized for the logarithm of the random effects variance (refer to the Appendix C for complete details). It should be noted that the analytical framework employed here is substantially more flexible than the conventional negative binomial regression commonly used in RNA-seq count data analysis [37]. Furthermore, it diverges from the *t* test-based analysis that generated the training sample depicted in Figure 1.

In this experiment, we consider m=10,512 genes and only $n_{x}=n_{y}=3$ patients under each condition, making a total of six patients. For every gene *i*, the unscaled Bayes factor, $B_{i}$ , is computed separately to evaluate the model incorporating tissue effects against the null model devoid of such effects. Both models incorporate patient-specific random effects. Using the trained Convolutional Neural Network (CNN) based on the data shown in Figure 1, we report in Table 1 the probabilities and genes that could be linked to carcinoma tissue. These results are presented in conjunction with the results obtained by the BH procedure, which tests the significance of the tissue effect coefficient.

Most of the remaining genes exhibit a probability of less than 50% of relevance to the condition under study. In particular, 658 genes produced adjusted *p*-values less than 0.001 according to the BH procedure, which is a very noisy result.

Interestingly, according to the proposed procedure, only the TTN and KRT genes had previously been identified in the study by [37]. Other genes such as SPRR [38], NEB [39], ITGB [40], and PLEC [41] have been subsequently associated with tumor conditions in the cited literature. This observation underscores the valuable insights that could be gleaned from the data if analyzed using our proposed approach. In contrast, genes highlighted by the BH method, such as PTHLH, COL4A6, and PTGFR, have only tangential associations with tumors. For example, PTHLH has been discussed in the context of cow tumors [42], again suggesting that the BH method may produce noisy results compared to the proposed one.

Unlike the situation described in Section 4.2, the ground truth in this case is unknown. However, our objective is to demonstrate that the proposed CNN methodology outperforms the BH approach. This is particularly noteworthy given that the Bayesian Poisson sampling model (see Appendix C) diverges substantially from the model used to generate Figure 1. This argument is further substantiated by an additional simulation study detailed in Appendix D. The suboptimal performance of the BH method is attributable to the misspecification of the negative binomial model [37] due to the inclusion of patient-specific random effects. Furthermore, the presence of a nuisance dispersion parameter adversely affects the reliability of *p*-values, since these are no longer calibrated with respect to the U(0,1) distribution, thus compromising the efficacy of multiple hypothesis testing procedures such as BH [3].

### 4.4. Example RNA-Seq 2: Normal vs. Tumor Tissue

We reviewed the RNA-Seq data of Arabidopsis thaliana as discussed in [43]. The data set focuses on the plant’s response to the bacterium Pseudomonas syringae, a model organism for studying plant-pathogen interactions. The purpose of the analysis is to identify differentially expressed genes that illuminate how plants defend themselves against such pathogens.

Three Arabidopsis plants, each six weeks old, were treated with Pseudomonas syringae, while control plants were given a mock pathogen. Subsequently, total RNA was extracted from the leaves, resulting in three independent biological replicates. Each set, comprising $n_{x}=n_{y}=3$ RNA samples, was sequenced and RNA-Seq counts were collected for m=13,930 genes. Samples are not independent, and this requires an adjustment for time-dependent effects.

The Bayesian regression model used is analogous to the one described in Section 4.3. However, an additional layer of complexity is introduced by incorporating a first-order autoregressive process to account for temporal random effects (see Appendix E). As before, the BFs are unscaled due to improper priors on the model coefficients.

Our results, detailed in Table A1 of the Appendix F, highlight only 31 genes with a probability greater than 0.5 of having an interaction effect with Pseudomonas syringae. This contrasts starkly with the 387 genes identified in the original study by [43], which employed the *q*-values [33] to control FDR at 5%. The BH method identified as many as 1805 genes, reinforcing the notion of an inflated Type I error because of maybe-dependent tests. In particular, the FDR control using *q*-values has been shown to converge to the BH control [33] asymptotically, validating our use of the BH method as a benchmark. Interestingly, all but two of the 31 genes were previously reported in [43]. The two exceptions, AT4G12800 and AT1G54410, have been implicated in the response of the pathogen in subsequent studies [44,45].

## 5. Remarks

We alert the reader to three critical limitations associated with the use of CNNs, which also constitute the main theoretical drawbacks of this study: (*i*) The output of the neural network does not come with associated uncertainty measures; ( ii ) Due to its complex architecture and predictive focus, an exact interpretation of the trained CNN is elusive, although some insights can still be gleaned; ( iii ) Like any statistical model, the efficacy of an NN rests on the (untestable) assumption on the sampling model for tests that makes the training and testing samples exchangeable.

Looking at the usage of BFs in this work, we may think that there always exist proper priors that make $cB_{i}$ , a proper BF for test *i* interpretable in the sense of providing the evidence for test *i* as suggested by Jeffreys, which is true but at the cost of introducing arbitrary *c* in the term. Then $cB_{i}$ is interpretable, but it is also arbitrarily interpretable as a measure of evidence due to the presence of *c*. In contrast, in the definition of *W*, based on the proper BFs $cB_{i}$ , *c* simplifies and the same occurs when ordering tests according to the BFs $cB_{i}$ . The problem of finding a cutoff on $cB_{i}$ is exactly that of fitting a CNN that allows one to fix a cutoff on the scale of $\hat{p}$ that considers the multiplicity of tests, as do some MHT procedures with adjusted *p*-values. We claim that the proposed scale on which $\hat{p}$ lies is much more interpretable as the direct probability that the test is observed under the null or alternative given the evidence from all tests.

The current methodology can also be implemented without explicit Bayesian computations. In particular, BFs, denoted $B_{1},\ldots ,B_{m}$ , can be substitute calibrated *p* values according to the lower bound of the BF as elaborated in [46,47]. Specifically, for all $pv_{i}<\exp\left(-1\right)$ , the infimum of the BF for the *i*th test can be expressed as

(6) $$cB_{i}\geq B_{i}=\left\{\begin{array}{cc} {\left[-epv_{i}\ln\left(pv_{i}\right)\right]}^{-1}, & \mathrm{for}\;pv_{i}<\exp\left(-1\right)\\ 1, & \mathrm{otherwise} \end{array}\right.,$$

where *c* symbolizes the calibration constant for the unknown true BF $cB_{i}$ . This uncalibrated BF can then be utilized in our method after scaling the computed *p*-values by Equation (5). In the end, this allows us to generally extend the use of the proposed method to statistical analyses that are not per se Bayesian.

Our strategy relies on a singularly trained CNN. Future work could explore alternative architectures, such as bidirectional CNNs [48]. However, our existing CNN demonstrates remarkable performance in various settings, almost achieving an AUC close to 1, irrespective of the underlying sampling model specifically considered.

This innovative application of transfer learning [49] to MHT serves as the cornerstone of this study. The approach draws parallels with classical statistical techniques like the use of the Central Limit Theorem but within a computational context. The primary advantage lies in reusing CNN weights trained on one dataset (simulated and observed from calibration studies), as seen in Figure 1, to analyze different MHT problems. This approach essentially mirrors the untestable assumptions used in the BH procedure, such as positive regression dependence [1,50].

In summary, our results are promising for broader adoption of CNN-based strategies in MHT, especially given that the network performs consistently across divergent testing frameworks (e.g., *t*-tests, mixed-effect models, dependent/independent tests).

## Figures and Tables

**Table 1:** Probability of genes associated with Squamous cell carcinomas according to the CNN trained with *W* in Figure 1 and the BH procedure.

Gene:	TTN	KRT13	SPRR3	NEB	KRT4	ITGB4	PLEC
CNN, $\hat{p}$ :	0.999	0.890	0.813	0.788	0.762	0.700	0.582
**Gene:**	**PTHLH**	**PTHLH**	**PTHLH**	**PTHLH**	**COL4A6**	**PTGFR**	**PTGFR**
BH:	$8\cdot 10^{-17}$	$1\cdot 10^{-16}$	$1\cdot 10^{-16}$	$2\cdot 10^{-16}$	$8\cdot 10^{-15}$	$5\cdot 10^{-15}$	$4\cdot 10^{-15}$

**Figure A1:**
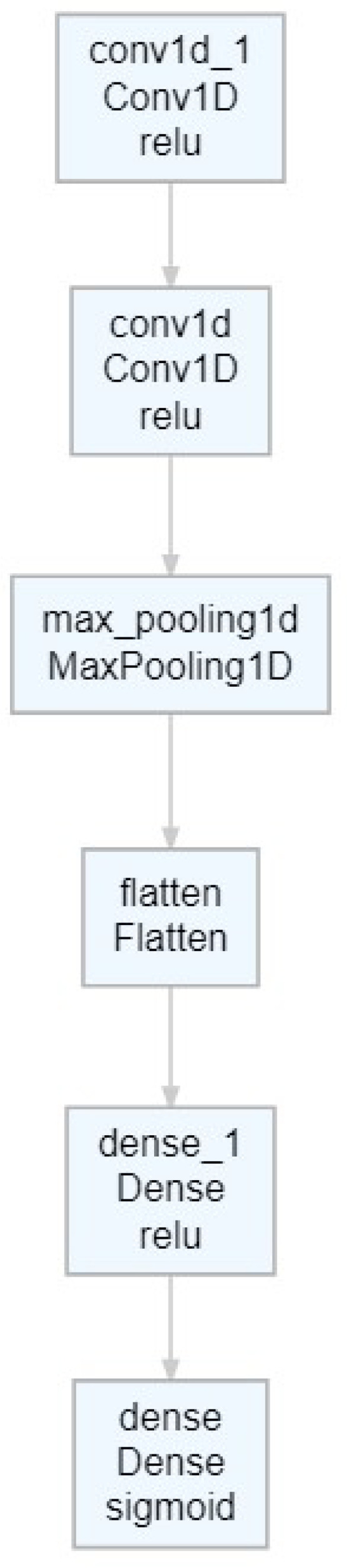
Schematic representation of the used CNN architecture by means of the sequence of layers and their type.

**Figure A2:**
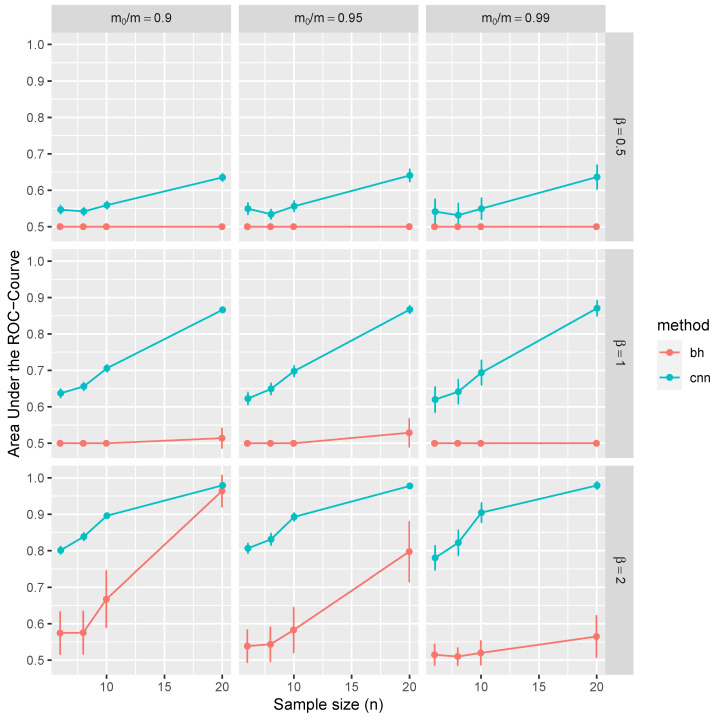
For 100 MHT simulations under the Poisson model with random effect and a given configuration, we consider the average area under the ROC curve (vertical) along with 99.9% confidence intervals for independent tests, under different simulations scenarios varying according to the samples sizes *n*, proportion $m_{0}/m$ of tests under the null (horizontal) and the Poisson regression coefficient of the effect under the alternative (right vertical scale). Averages are reported for the BH procedure on *p*-values from likelihood ration tests (red) and the proposed CNN on *W* (blue).

## Data Availability

Data set for training the CNN have been generated with the code available at https://colab.research.google.com/drive/1TdM1FSVKm1GI55riUXoLbzcEcM3FeoNg?usp=sharing. Data for Example RNA-seq 1 are available from CRAN package edgeR and for Example RNA-seq 2 are available from CRAN package NBPSeq::arab.

## Abbreviations

    The following abbreviations are used in this manuscript:

BF	Bayes Factor
CNN	Convolutional Neural Network
DL	Deep Learning
MHT	Multiple Hypothesis Testing
FDR	False Discovery Rate
FNR	False Nonrejection Rate
ROC	Receiver Operating Characteristic

## Appendix A. The Unscaled Bayes Factor for Testing Two Normal Means

Suppose the usual two-group model with *m* features and denote by $x_{m\times n_{x}}$ the outcome in group *X* with $n_{x}$ replications and $y_{m\times n_{y}}$ the outcome in group *Y* with $n_{y}$ replications. Let $X_{i}∼N\left(\mu_{X_{i}},\sigma_{X_{i}}^{2}\right)$ and $Y_{i}∼N\left(\mu_{Y_{i}},\sigma_{Y_{i}}^{2}\right)$ for i=1,2,…,m . The set of hypotheses for $\sigma_{X_{i}}^{2}>0$ , $\sigma_{Y_{i}}^{2}>0$ unknown, is the following, for i=1,…,m :

$$H_{0i}:\mu_{X_{i}}=\mu_{Y_{i}}=\mu_{i}\mathrm{versus}H_{1i}:\mu_{X_{i}}\neq \mu_{Y_{i}},\;\forall \sigma_{X_{i}}^{2}>0,\;\forall \sigma_{Y_{i}}^{2}>0.$$

With the usual default priors:

$$\begin{array}{ccc} \pi_{0}\left(\mu_{i},\sigma_{X_{i}}^{2},\sigma_{Y_{i}}^{2}\right) & \propto & \sigma_{X_{i}}^{-2}\sigma_{Y_{i}}^{-2}\cdot 1_{R\times R^{+}\times R^{+}}\left(\mu_{i},\sigma_{X_{i}}^{2},\sigma_{Y_{i}}^{2}\right),\\ \pi_{1}\left(\mu_{X_{i}},\mu_{Y_{i}},\sigma_{X_{i}}^{2},\sigma_{Y_{i}}^{2}\right) & \propto & \sigma_{X_{i}}^{-2}\sigma_{Y_{i}}^{-2}\cdot 1_{R\times R\times R^{+}\times R^{+}}\left(\mu_{X_{i}},\mu_{Y_{i}},\sigma_{X_{i}}^{2},\sigma_{Y_{i}}^{2}\right), \end{array}$$

the unscaled BF for $H_{1i}$ versus $H_{0i}$ is (see [9,10] for further details).

(A1)
$$B_{i}=\frac{Beta\left(\frac{n_{x}-1}{2},\frac{1}{2}\right)Beta\left(\frac{n_{y}-1}{2},\frac{1}{2}\right)\sqrt{S_{X_{i}}^{2}S_{Y_{i}}^{2}}}{\int_{\mu_{i}\in R}{\left(1+{\left({\bar{X}}_{i}-\mu_{i}\right)}^{2}/S_{X_{i}}^{2}\right)}^{-\frac{1}{2}n_{x}}{\left(1+{\left({\bar{Y}}_{i}-\mu_{i}\right)}^{2}/S_{Y_{i}}^{2}\right)}^{-\frac{1}{2}n_{y}}d\mu_{i}},$$

where Beta(a,b) is the beta function evaluated in a,b and ${\bar{X}}_{i},{\bar{Y}}_{i},S_{X_{i}}^{2},S_{Y_{i}}^{2}$ are sample means and variances for group *X* and *Y*, respectively. The Beta() functions comes by the ratio of corresponding Gamma functions that arises after integrating the two variances $\sigma_{X_{i}}^{2}$ , $\sigma_{Y_{i}}^{2}$ and the common mean $\mu_{i}$ under $H_{0i}$ .

## Appendix B. Architecture of the Actual CNN

CNN used in the paper is made up of the following layers of nodes indicated in the order from the input nodes to the output node:

1. the input node of the sequences *W*s of length ten tests (we have m/10 training samples). The length of these sequences, also known as the *batch size* affects mainly the fitting process rather than the final performance in the classification tests. This is so, although it is worth noting that smaller batch sizes often lead to better generalization on unseen tests. This is in part due to the fact that smaller batches introduce more noise during training, which acts as a form of regularization of the classification model.
2. Two sets of convolution layers with kernels of size k=4 . The first has 64 filters, and the second has 32. With only one convolutional layer before pooling, the network captures the basic features of the test sequence, such as the eventual dependency on the sequence and the differences between *W* under the null and under the alternative. These features are typically more generalized and less refined. However, two convolutional layers allow the network to learn more complex and abstract features, especially around the separation point $\hat{i}$ .
3. One max-pooling node, which returns the maximum of the input coming from the feature map every two features. These nodes implement the translation invariance property mentioned in Section 2.2, but aggregate and give weights to all the features captured in the above layers that are relevant to determine whether *W*s have been observed before or after the separation point $\hat{i}$ . This node implements the hierarchy of collected features in the above nodes, mimicking the hierarchical modeling approach typical in Bayesian statistics.
4. One set of 36 dense layers (e.g., all connected nodes). While convolutional and pooling layers are adept at local randomness behavior in the sequence of tests represented by *W*s, dense layers help to make sense of the estimated complex patterns and relationships among local behaviors to be used for classifying tests.
5. The output node, which is the logistic function that returns the probability $\hat{p}$ for each test to belong to the alternative. This node is needed to have an estimation of the probability that a test comes from the null or alternative hypothesis.

To summarize the above architecture, Figure A1 reports it.

All nodes, except the last one, use the rectified linear unit activation function, which returns the input if positive and zeros otherwise. The loss function is the binary cross-entropy (e.g., the log density of a Bernoulli distribution), and the optimizer is the Adam one. This optimizer is an extension of the stochastic gradient descent algorithm embedded in the backpropagation algorithm used to optimize the weights of the NN [14]. There are a total of 10,921 parameters (i.e., weights) to train, and most of them will be just zeros, as the CNN-induced model is far too complex to be learned with the proposed values of *m*.

## Appendix C. Poisson Regression with Random Effects

Let $Y_{jr}$ be the observed counts/abundance of RNA sequences for a given gene in the patient *r* at measurement/replication *j*. The Bayesian model is the standard Poisson regression with normal random effects for patients:

$$\begin{array}{ccc} Y_{jr}|\beta ,u_{r},X_{j} & ∼ & Poisson(\lambda \left(\beta ,u_{r},X_{j}\right))\\ log(\lambda \left(\beta ,u_{r},X_{j}\right)) & = & \beta^{T}X_{j}+u_{r}\\ \beta & ∼ & \pi \left(\beta \right)\propto 1,for\;\beta \in R^{col(X_{j})}\\ u_{r}|\tau & ∼ & Normal(0,\tau )\\ \tau & ∼ & Gamma(1,0.00005), \end{array}$$

where Poisson( λ ) is the Poisson distribution with mean λ and Gamma( a,b ) is the Gamma distribution with mean a/b . The design matrix *X* includes the intercept column and the tissue column only in model $H_{1}$ . For comparison of the two models $H_{0}$ (not including the tissue effect in the design matrix) and $H_{1}$ , the BF is unscaled due to a flat improper prior on the vector of coefficients β . The model has been fitted using INLA [51]. INLA returns the marginal approximations under the alternative and the null model, $m_{i1}\left(\cdot \right)$ and $m_{i0}\left(\cdot \right)$ , respectively. Thus, the relationship between these two numbers gives the unscaled BF, $cB_{i}$ .

## Appendix D. Comparison of CNN versus BH in Poisson Regression Random Effects

Let β be the coefficient of the effect of the treatment/tissue under test according to the model described in Appendix C. We simulate 100 MHT experiments, each with m=1000 tests, for each combination of

- different signals in the tests from the alternative: $\beta_{i}=0.5,1,2$ for $i\in H_{1}$ and $\beta_{i}=0$ for $i\in H_{0}$ ;
- sample sizes *n* for balanced by subjects/patients designs, with two subjects/patients: n=6,8,10,20 (where n/2 samples are from each subject).

In each test, patients’ random effects are drawn from a standard normal distribution; specifically, there are two draws for each test since there are only two subjects.

The results are analyzed with the model detailed in Appendix C and with the Negative Binomial regression. In both cases, the tests refer to the tissue coefficient. The results of the BFs of the model in Appendix C are analyzed with the CNN already fitted to the training sample in Figure 1. Finally, the BH procedure is applied to the *p*-values from the likelihood ratio tests of the binomial regression model (that is, the alternative model has the tissue effect and the null has only the intercept). This model is a reference for these types of experiments.

Figure A2 reports the average AUC over 100 replications along with 99.9% confidence intervals.

We can see that the proposed CNN procedure performs better than the BH one even if the model in Appendix C radically differs from that used to produce Figure 1. The BH performs poorly as the negative binomial model [37] is a misspecified model due to the patient’s random effects. This case is an example in which the values *p* are not calibrated, which affects the MHT procedure as the BH procedure [3].

## Appendix E. Poisson Regression with Autoregressive Random Effects

Let $Y_{jr}$ be the observed counts/abundance of RNA sequences for a given gene at time *r* at measurement/replication *j*. The Bayesian model is the Poisson regression with time autoregressive random effects of order 1:

$$\begin{array}{ccc} Y_{jr}|\beta ,u_{r},X_{j} & ∼ & Poisson(\lambda \left(\beta ,u_{r},X_{j}\right))\\ log(\lambda \left(\beta ,u_{r},X_{j}\right)) & = & \beta^{T}X_{j}+u_{r}\\ \beta & ∼ & \pi \left(\beta \right)\propto 1,for\;\beta \in R^{col(X_{j})}\\ u_{1}|\tau ,\rho & ∼ & Normal(0,\tau \left(1-\rho^{2}\right))\\ u_{r}|u_{r-1}\tau ,\rho & ∼ & Normal(\rho u_{r-1},\tau ),for\;r>1\\ \tau (1-\rho^{2}) & ∼ & Gamma(1,0.00005)\\ \log\left(\frac{1+\rho }{1-\rho }\right) & ∼ & Normal(0,0.15) \end{array}$$

where Poisson( λ ) is the Poisson distribution with mean λ and Gamma( a,b ) is the Gamma distribution with mean a/b . In this $\tau (1-\rho^{2})$ is the marginal precision which is gamma distributed to explode the semi-conjugacy with the normality of *u*s. Parameter $\log\left(\frac{1+\rho }{1-\rho }\right)$ recalls the usual logarithmic odds on which a normal prior distribution is usually employed because a posterior kernel made of normal density is much more conveniently approximated under the INLA approach. The design matrix *X* includes only the intercept and pathogen columns in the model $H_{1}$ . To compare the two models $H_{0}$ (without pathogen column) and $H_{1}$ , the BF is not scaled due to an improper flat prior on the coefficient vector β . The model was fitted using INLA.

## Appendix F. Genes Related to the Reaction to Pseudomonas Syringae

According to the CNN fitted on the training sample in Figure 1 and applied to the sequence of *W* induced by $B_{i}$ of the model illustrated in Section 4.4, the genes reported in Table A1 have more than 50% probability of being related to the defense of Arabidopsis plants against Pseudomonas syringae.

**Table A1:** Probability of genes associated with defending Arabidopsis plants to Pseudomonas syringae according to the CNN trained with *W* in Figure 1. Genes with * have not been reported in [43] but in other studies.

Gene	$\hat{p}$	Gene	$\hat{p}$
AT1G76930	1.00	AT5G13220	0.75
AT4G22470	0.99	AT5G37600	0.74
AT4G12500	0.94	AT3G22120	0.74
AT2G45180	0.86	AT4G30190	0.73
AT5G64120	0.85	AT3G63160	0.72
AT2G43620	0.82	AT1G02930	0.70
AT4G38770	0.80	AT1G65845	0.69
AT5G54160	0.80	AT1G02920	0.69
AT2G10940	0.80	AT4G12470	0.68
AT1G67090	0.80	AT4G12480	0.67
*AT1G54410	0.80	AT3G46280	0.65
AT2G39200	0.79	AT4G10340	0.63
AT1G29930	0.77	*AT4G12800	0.63
AT4G12490	0.76	AT3G26740	0.58
AT5G54770	0.76	AT1G09560	0.56
